# Component-Resolved Diagnosis in Food Allergies

**DOI:** 10.3390/medicina55080498

**Published:** 2019-08-18

**Authors:** Elisabetta Calamelli, Lucia Liotti, Isadora Beghetti, Valentina Piccinno, Laura Serra, Paolo Bottau

**Affiliations:** 1 Pediatric and Neonatology Unit, Imola Hospital, 40026 Imola, Italy; 2 Pediatric Unit, Civic Hospital, 60019 Senigallia, Italy; 3 Pediatric Unit, Department of Medical and Surgical Sciences (DIMEC), S.Orsola-Malpighi Hospital, University of Bologna, 40138 Bologna, Italy

**Keywords:** alfa-gal, casein, gliadin, lipid transfer protein, ovomucoid, parvalbumin, tropomyosin

## Abstract

Component-resolved diagnostics (CRD) in food allergies is an approach utilized to characterize the molecular components of each allergen involved in a specific IgE (sIgE)-mediated response. In the clinical practice, CRD can improve diagnostic accuracy and assist the physician in many aspects of the allergy work-up. CRD allows for discriminatory co-sensitization versus cross-sensitization phenomena and can be useful to stratify the clinical risk associated with a specific sensitization pattern, in addition to the oral food challenge (OFC). Despite this, there are still some unmet needs, such as the risk of over-prescribing unnecessary elimination diets and adrenaline auto-injectors. Moreover, up until now, none of the identified sIgE cutoff have shown a specificity and sensitivity profile as accurate as the OFC, which is the gold standard in diagnosing food allergies. In light of this, the aim of this review is to summarize the most relevant concepts in the field of CRD in food allergy and to provide a practical approach useful in clinical practice.

## 1. Introduction

IgE-mediated food allergy is defined as “an adverse reaction to food mediated by an immunologic mechanism, involving specific IgE (sIgE)” that results in high morbidity and even in life-threatening reactions [1]. Its worldwide prevalence varies from age and country of residence. Otherwise, while the rate of self-reported food adverse reactions is up to 17%, the real prevalence of a food allergy confirmed by an oral food challenge (OFC) is estimated to be around 1% [1]. 

The conventional diagnostic work-up for IgE-mediated food allergy begins with the clinical history, followed by in vivo and/or in vitro tests (Skin Prick Test (SPT) and/or specific IgE (sIgE) test) against the whole allergen source and usually ends with the OFC, which still remains the gold standard in diagnosing food allergies [2]. Extracts used for SPT and/or IgE testing are composed of many components, the majority of which are not significant for the diagnostic process. [2]. To overcome these limits, many allergens have been purified in the last few decades and are available as recombinant or native molecular proteins both for conventional sIgE antibody assays (ImmunoCAP Allergen Components) and microarray platforms (ImmunoCAP ISAC, FABER test) [3]. 

Component-resolved diagnostics (CRD), also called component resolved diagnosis is a diagnostic approach that utilizes these purified native or recombinant allergens to detect the sIgE antibodies response against the individual allergenic molecules [4]. In the diagnostic pathway of food allergy, this technique aims to characterize the molecular sensitization profile of a food allergic patient, with the goal to improve the specificity of sIgE testing for the selected foods [5]. CRD can allow for the discrimination of genuine sensitization from sensitization due to cross-reactivity [5]. Moreover, it can be useful to stratify the clinical risk associated with a specific sensitization pattern and predict the outcome of the OFC [2]. However, although CRD was developed to improve the diagnostic accuracy of sIgE-based assays, some cutoffs have been proposed to identify children who will react to specific allergens. The specificity and sensitivity levels are not accurate enough to replace the OFC, which is the gold standard in the diagnosis of food allergies [2]. On the other hand, this approach requires and adequate interpretation, to avoid unnecessarily prescribing elimination diets and adrenaline auto-injectors, which may have a subsequent negative repercussion on the quality of life of patients. 

### Aims and Methods

The aim of this review is to summarize the most recent relevant concepts in the field of CRD, specifically pertaining to food allergy. Further, this study aims to provide a practical approach useful in the clinical practice. For the complexity of the topic, we gave priority to the most common allergens implicated in IgE-mediated reactions in childhood. References were identified by PubMed searches dating up to May 2019 that used the following search terms: “component resolved diagnosis”, “food allergy”, and “children”. For allergen nomenclature, we followed the “Nomenclature system for allergens” that was recommended by the International Union of Immunological Societies (IUIS) [6,7]. This review doesn’t meet the criteria of a systematic review. 

## 2. Cow’s Milk

Cow’s milk (CM) is one of the main causes of food allergy and of anaphylaxis in childhood [1]. CM and dairy products are among the main source of proteins, calories, and calcium for infants and young children. Cow’s milk protein allergy (CMPA) prevalence ranges between 1.8% and 7.5% in the first year of life [1]. CMPA diagnosis is usually the result of a suggestive clinical history and sIgE and/or SPTs. Many kinds of proteins that have different features are present in CM (Table 1). More than 50% of the individuals with CMPA are sensitized to caseins, beta-lactoglobulin, and alpha-lactalbumin, which are major CM allergens. Most CMP allergic patients are sensitized to both caseins and whey proteins [8,9]. Proteins in CM are a class 1 food allergen; they survive in the gastrointestinal tract and may elicit allergic sensitization and systemic reactions if ingested [10]. 

Different sensitization profiles to CM allergens have been documented in the general population. IgE-sensitization to caseins, beta- lactoglobulin, and alpha-lactalbumin is strongly correlated; otherwise, IgE-sensitization to bovine serum albumin (BSA) is not related to other CM proteins and may find a cross-reactivity with beef [11]. The tolerance development in CMPA can be followed-up with molecular diagnosis.

A prospective study shows that children affected by CMPA with lower serum levels of sIgE to CM (alpha-lactalbumin, beta-lactoglobulin, kappa-casein, and alpha s1 casein) had better possibilities of eventually become tolerant to CM [9]. IgE epitope-binding patterns were constant in patients with persisting CMA; development of tolerance to CM is related to the decreased epitope binding by IgE and associated increase in corresponding epitope binding by IgG4 [12]. In order to predict which patients will develop tolerance to CM, monitoring casein-specific and beta-lactoglobulin-sIgE concentrations and IgE/IgG ratios can be useful [13].

The allergenicity of CM protein is modified by extensive heating e.g., baking [14]. Caseins are more resistant to heating compared to whey proteins. Heating reduces allergenicity of beta-lactoglobulin through the formation of the intermolecular disulphide bonds and binding to other food proteins [15]. Extensively heated CMP are usually tolerated by children with mild IgE-mediated CMPA. On the contrary, there is a higher risk for anaphylaxis and more persistent CMPA in children who react to baked milk. IgE antibodies directed against sequential CMP epitopes (especially casein) are mainly produced by children with more persistent CMPA, while children who tolerate baked milk mainly generate IgE antibodies against conformational CMP epitopes (destroyed by high temperature) [16]. Lack of tolerance to baked milk can be predicted by high levels of sIgE antibodies directed against casein [17,18]. Implementation of baked milk products into the diet of children with CMPA seems to accelerate tolerance to CM [19,20].

Moreover, dosage of sIgE antibodies can be used when identifying patients at risk of severe adverse reactions to milk oral immunotherapy (OIT). The literature suggests that the detailed analysis wherein IgE and IgG4 binds to CM peptides might predict a response to milk OIT and increase the safety of CM OIT [21,22]. 

CMPA have a favorable prognosis and the majority of children become tolerant in school age [23]. However, CM-sIgE greater than 50 kUA/L are associated with persistent CMPA until adolescence or adulthood [24]. When diagnosing CMPA, CRD is not superior to conventional diagnostic tests based on the whole allergen extracts [25]. Still, CRD can be useful in diagnosing tolerance to extensively heated milk proteins and may predict the natural course of CMPA and the response to milk OIT.

## 3. Hen Eggs

Allergy to hen eggs is one of the most common IgE-mediated food allergies in children; it affects 1–2% of children [26] and is phenotypically heterogeneous and potentially life-threatening. Several phenotypes of egg allergy have been recognized, including those who tolerate extensively heated egg in bakery products. 

Egg proteins have been identified both in egg white than in yolks [27].

*Gal d 1* to *5* are five proteins that are most commonly involved in allergic reactions to hen eggs (Table 2). Ovomucoid is present in lower quantity in hen eggs white than ovalbumin; nevertheless, it is probably the immunodominant hen eggs allergen. 

The primary and recommended diagnosis and treatment of egg allergies in children is usually egg white IgE testing. Egg white extract combines ovomucoid and ovalbumine, which are the most common major allergens, and represent the most accurate test for the initial diagnostic step [28].

Several authors have suggested the use of cutoff values to obtain a diagnosis of egg allergy without performing an OFC. Despite this, none of the cutoff values by themselves allow a firm diagnosis of egg allergy. Further studies are needed to determine the diagnostic cutoff of sIgE and SPTs for heated and baked egg allergy [29,30,31].

In order to obtain a fine-tuned diagnosis of egg allergy, molecular diagnosis can be helpful, especially for characterizing different clinical situations:

(a) patients are sensitized to hen eggs, but are clinically tolerant, with a positive serum IgE test to hen egg whites, usually in a low to midrange value and negative or low serum IgE test to ovomucoid;
(b) patients who tolerate cooked eggs or processed foods containing cooked eggs. These patients have IgE tests similar to the previous cases. Serum sIgE to ovalbumin might be elevated in a similar range than the test to egg white;
(c) patients presenting allergy to all forms of egg (raw and baked). Serum sIgE to egg white are often in the middle to upper range in these patients. Moreover, serum sIgE to ovomucoid and ovalbumin can be elevated.

In addition, some children have shown to tolerate extensively heated egg. Extensively heating egg seems to decrease its allergenicity; 64% to 84% of children allergic to eggs have been found to tolerate baked-egg products [32]. Children with an IgE-mediated hen egg allergy often tolerate baked egg within a wheat matrix [33]. Initiation of a baked egg diet seems to accelerate the development of regular egg tolerance compared with strict avoidance. Accurate predictors of natural tolerance development to cooked and uncooked eggs have not been identified in egg-allergic patients.

The Ovomucoid *Gal d 1* IgE reactivity appears to be a predictor of egg clinical allergy. A high frequency of egg allergy is evidenced in *Gal d 1* positive children, whereas *Gal d 1* negative children seem to better tolerate boiled eggs [34]. 

Although the ovomucoid sIgE level may be helpful in predicting cooked egg challenge outcomes, some studies did not support a role for ovomucoid sIgE replacing egg white sIgE testing in the evaluation of egg allergies [35]. 

The literature suggests that starting with IgE measurement to egg white, followed by IgE to ovomucoid, will significantly increase the sensitivity of diagnostic testing compared to testing egg white only, although it does decrease specificity [36]. 

Additionally, patients with conformational epitopes to hen eggs are more likely to resolve their allergy compared with those with IgE binding to sequential epitopes [37]. 

Ovalbumin-specific IgG4 is an independent predictor of tolerance development to raw egg. Ovalbumin-sIgE/sIgG4 ratio, followed by SPT, is useful when identifying patients with high probability to tolerate cooked and uncooked eggs [38].

Murine models of baked egg diets demonstrate that heated egg can lead to protection against anaphylaxis and cause immune changes. These results have been confirmed by most observational human studies of baked egg diets, which demonstrated clinical resolution of allergy and favorable immune changes, especially if compared to controls. In any case, some studies in the literature do not confirm the immune-modifying effect of the baked egg diet [39]. Physician-supervised introduction of baked milk and egg is recommended because systemic symptoms until anaphylaxis can occur [40]. Diagnosis and monitoring for resolution often requires OFC, which can result in anaphylaxis. The CRD approach, microarray analysis, and epitope mapping are being evaluated to determine if there is a need to replace or reduce OFCs [2,5]. 

Nowadays, the first diagnostic test should be represented by the measurement of serum IgE or SPT testing of egg whites. Further, it should be available to primary care physicians.

The use of molecular components is the most helpful method to define tolerance to cooked eggs, even if more studies are necessary to confirm the clinical utility of such tests [2]. Despite this, it is important that the use and the interpretation of these tests is conducted by allergy specialists who carefully consider the clinical history of the patient [2]. 

## 4. Soy

The pathogenesis of soybean allergies in the pediatric age—in particular, those children with CMA who use soy based formula as a substitute of CMP—is due to the primary sensitization through the gastrointestinal tract [3,41,42,43]. Soybean is a legume and consumed whole as a processed food. Further, it may be added in many industrial foods as a hidden allergen [3]. Until now, at least 16 allergens have been identified in soy [2,7] (Table 3). Among them, *Gly m 5* (7S Globulin), *Gly m 6* (11S Globulin), and *Gly m 8* (2S albumin) are considered to be major soy allergens belonging to the class of Seed Storage Proteins (SSP) [41,42,43]. These allergens are considered as markers of primary sensitization and characterized by a high stability both to the heat exposure and to the gastric digestion, which are implicated in severe systemic reactions [41,42,43]. Meanwhile, Gly m 4, a pathogenesis related class 10 protein (PR-10) belonging to the Bet v 1 homologous family, is characterized by low stability and commonly associated with oral allergy syndrome (OAS) [5,44]. Moreover, this allergen is considered implicated in allergic reactions to moderately processed soy powder in birch pollen allergic patients. Despite this, the combination of *Gly m 4* sensitivity and the intake of large amounts of mildly processed soy, such as soy drinks, can induce severe reaction in birch pollen-allergic individuals [45]. 

## 5. Peanuts, Tree Nuts, and Seeds

Peanut and tree nut allergies are characterized by IgE-mediated reactions to nut proteins. There are two clinical phenotypes of nut type I reactions: a primary nut allergy, characterized by systemic and often severe reactions to nuts, and pollen food syndrome (PFS), also known as oral allergy syndrome (OAS), which is characterized by seasonal allergic rhinitis and a history of mild oropharyngeal symptoms in response to fresh fruit, vegetable, or nut ingestion [46,47]. Primary nut allergies arise most commonly in the first five years of life [48]. Nut allergy prevalence varies according to differences in populations examined, study design, and diagnostic criteria [47]. Reported prevalence of peanut allergies varied from 0.5% to 2.5%, whereas tree nut allergies varied from from 0.2% to 2.2% [49]. Nut allergies tends to cause severe reactions and usually persist over time. The majority of severe non-fatal and fatal accidental reactions occurs in teenagers and young adults; allergic reactions to nuts may be more severe in adults than children [50]. Nut allergies (peanuts or tree nuts) is the main cause of anaphylactic death in adolescents and young adults [51]. It is noteworthy that a clinical history of asthma in food allergy increases the risk of a severe allergic reaction [52]. 

A suggested algorithm for diagnosing nut allergies relies on a patient’s clinical history. An unequivocal history of an immediate reaction following the ingestion of a peanut or tree nut, with positive tests for sIgE, is usually sufficient to establish the diagnosis for suspected IgE-mediated reactions [47]. Either SPT or serum specific total nut IgE test are usually performed. The magnitude of a SPT or sIgE is correlated to the probability of clinical allergy but does not relate to clinical severity [47]. 

CRD allows for an increased diagnostic accuracy and for assessing the risk and type of reaction [1,2] (Table 4). *Ara h* 2 is the major peanut allergen and *Ara h* 2 sIgE can discriminate between allergic and tolerant children better than total peanut sIgE [53]. Several studies have established cutoff values for the peanut component *Ara h* 2. The reported predictive value of *Ara h* 2 varies amongst different populations. Measurements of *Ara h* 1, 3, and 6 appears less useful. However, if peanut sIgE is positive and sIgE *Ara h* 2 is negative, then other peanut components can be useful in combination with the clinical context. In contrast, isolated sensitization to *Ara h* 8 (PR-10 protein and birch pollen allergen Bet v1- homologue) is a marker of milder or local symptoms [54]. In southern Europe, for example, the Lipid Transfer Protein (LTP) (*Ara h* 9) may act as a marker of severity, as it is associated with systemic and more severe reactions [55]. Finally, patients with profilin or CCD sensitization to peanuts alone usually react with no or local oral symptoms and heated peanuts may be tolerated [2,5]. 

Sensitization to the hazelnut component, *Cor a* 9 and *Cor a* 14, are more specific for primary hazelnut allergies, especially when compared to hazelnut sIgE, with a certain variation amongst different populations in the predictive values of a sIgE level [56]. Sensitization to *Cor a* 9 and *Cor a* 14 has a strong impact on the distribution of hazelnut thresholds [57] and is a marker of more severe allergies [13]. Isolated sIgE to *Cor a* 1 (PR-10, Bet v 1 homologue) is often associated with clinical tolerance or mild, subjective oral symptoms, which suggests the possibility of PFS rather than a primary nut allergy [9]. Sensitization to PR-10 nut components in addition to seed storage components (e.g., *Ara h* 1, 2, 3, 6 or *Cor a* 9, 14) requires further evaluation of a patient’s history, as this suggests a diagnosis of a primary nut allergy [58]. Moreover, clinical reactions to nuts may reflect sensitization to non-specific LTP (e.g., *Ara h* 9, *Cor a* 8). This pattern of sensitization can be associated with both mild and severe systemic reactions [59]. Severe reactions in walnut-allergic patients are associated with SSPs (*Jug r* 1, *Jug r* 2) or LTP (*Jug r* 3) sensitization [60]. *Ana o* 3 appears to be the best predictor of cashew nut allergy, whereas, in children, sIgE to cashew components performs better than cashew-sIgE or SPT [61].

Despite sesame becoming a relevant allergen, very few studies regarding sesame allergy in childhood are available on CRD [62]. Moreover, studies on sesame-allergic patients showed that only a part of the allergenic proteins have been identified. Indeed, up to now seven allergens have been isolated. Among them, five are SSPs (*Ses i* 1, 2, 3, 6 and 7) and two oleosins (*Ses i* 4 and 5) [7]. In a pediatric population of 92 sesame-sensitized children, sensitization to r*Ses i* 1 (SSP) showed the same sensitivity to the sIgE against sesame (86.1% for r*Ses i* 1 vs. to 83.3% for the sesame), but a higher specificity (85.7% vs. to 48.2%) [63]. 

In addition to cutoff values predicting the probability of a positive OFC, other major information provided by CRD testing are useful to distinguish between primary anaphylactic and pollen-related food allergies, as well as shedding light on cross-reactivity and co-sensitization [64]. However, a recent systematic review on diagnostic accuracy and risk assessment of CRD for food allergies showed that few studies exist for each component and studies vary regarding the cutoff values used, which highlights the need of further research [65].

Since allergen sensitization does not necessarily imply clinical responsiveness, all sIgE tests including CRD should be evaluated within the framework of a patient’s clinical history [56,58]. A recent study showed that diagnosing food allergies based on suggestive symptoms and positive IgE tests was only in part confirmed by the gold standard provided by the food challenge [66]. 

Diagnostic food challenge is the gold standard to confirm or refute the diagnosis when history and sIgE test results are conflicting, in order to enhance diagnostic accuracy [47,67]. OFC to nuts may be required to make a definitive diagnosis when sIgE tests can only partially differentiate between serological cross-reactivity and co-sensitization versus clinical relevant cross-reactivity and co-allergy [67]. Therefore, OFC should be tailored to specific clinical situations in order to improve dietary and medical management [68]. 

## 6. Wheat

IgE-mediated reactions to wheat can occur after ingestion (food allergy), inhalation (occupational asthma/rhinitis; e.g., baker’s asthma), contact (contact urticaria), or physical exercise after eating wheat-based foods [wheat-dependent exercise-induced anaphylaxis (WDEIA)] [69]. The prevalence of wheat sensitization is around 4% in pre-school children [70] and increases from 2% to 9% from 2 to 10 years old, due to the secondary sensitization in patients with grass pollen allergy [71,72]. In contrast, primary wheat allergy arises in infancy, and in most cases resolves by 3 to 5 years of age [73]. Moreover, wheat allergy is estimated to affect up to 8% of children during the first three years of age and only 2% of adolescents and adults [74,75,76,77]. In contrast, baker’s asthma affects from 1 up to 10% of bakery workers, with a higher prevalence in males [78,79,80,81]. Finally, WDEIA typically affects adolescents and young adults, occurring after the ingestion of wheat-based products and subsequent physical exercise [82]. 

Until now, 28 allergenic components have been identified in wheat grain [2,7,83,84] (Table 5). The α-amylase/tripsin inhibitors (*Tri aA*_TI), the non-specific LTP *Tri a* 14, and the wheat serpin (*Tri a* 33) belong to the A/G fraction, while *Tri a* 19 (omega-5 gliadin) and the high and low molecular weight glutenins (*Tri a* 26 and *Tri a* 36) to the gluten fraction [2,84]. *Tri aA*_TIs are involved both in food allergy and in WDEIA. *Tri a* 14 is a relevant food allergen in Italian wheat allergic patients and is also associated with baker’s asthma, while *Tri a* 33 is involved both in food and respiratory wheat allergies [2,84]. Wheat gliadins are considered a marker of genuine wheat sensitization; in particular *Tri a* 19 is the major allergen in WDEIA and is also a relevant allergen in young children with immediate allergic reactions to ingested wheat [85]. *Tri a* 36 is a major allergen for patients with WDEIA and its expression increases during wheat seed maturation; its domain is resistant to heat and enzymatic digestion [86].

The allergy work-up in patients with suspected wheat allergies always includes an accurate history, investigating the tolerance to other cereals, the presence of pollen-induced respiratory allergies, the execution of in vivo tests (SPT to wheat), the detection of sIgE for the implicated allergens, and the available molecular components (wheat, gliadin, r*Tri a* 14, *Tri a* 19) [2]. 

## 7. Plant Foods (Fruits and Vegetables)

Fruit and vegetable are relevant allergens mostly in adolescents and adults. The identification of the allergens involved in cross-reactivity patterns has helped us understand the mechanisms of sensitization and how the allergen profiles determine different phenotypes [87]. Allergies to fruits and vegetables can be either a result of primary sensitization to food allergens through the gastrointestinal tract or the result of secondary sensitization to cross-reactive food allergens as a consequence of a genuine sensitization to homologous pollen or latex related allergens [88]. The most frequent clinical picture of fruit and vegetable allergies are pollen-food syndrome (PFS) and lipid transfer protein (LTP) syndrome [87]. 

PFS, also named oral allergy syndrome (OAS), is a hypersensitivity reaction to plant-based foods, which manifests most commonly with itching of the lips, tongue, and mouth. In contrast with other food allergies, OAS requires prior sensitization to a cross-reactive inhalant allergen rather than direct sensitization to a specific food protein [89]. The pollen proteins inducing an IgE–mediated reaction in OAS are structurally similar to proteins in some plat-derived foods. For instance, an allergy to grass may result in an allergic reaction following the intake of one or all of these foods in their raw form (e.g., melon, orange, tomato). Not every patient sensitized to pollen will develop this cross-reaction to PFS symptoms [90]. In brief, in PFS, fruit and vegetable allergies result from a primary sensitization to labile pollen allergens, such as PR-10 (*Bet v* 1 like allergen) or profilins (see below). The resulting phenotype is mainly mild, consisting of local oropharyngeal reactions. 

In contrast, LTP syndrome results from a primary sensitization to LTPs, which are stable plant food allergens, inducing frequent systemic reactions and even anaphylaxis [87].

Most of the fruits that causes adverse reactions (e.g., apple, peach, apricot, pear, strawberry, raspberry) belongs to the family of *Rosaceae* [91]. Fruits can be consumed both fresh and as processed products; allergenic molecules are contained both in the peel and in the pulp [2]. Data on the prevalence of fresh fruit allergies are scarce. In a systematic review of the overall prevalence of fruit allergies, Zuidmeer et al. estimated 0.1 to 4.3% [92]. Peaches induce most of the allergic sensitization in the general population (7.9%), followed by apples (6.5%) and kiwis (5.2%) [93]. As fruits, vegetables can enhance allergy symptoms in sensitized patients [94]. Vegetables belonging to the *Apiaceae* family (e.g., celery and carrot) are well known as potential allergic foods and are commonly consumed both cooked and raw [95]. 

As shown in Table 6, most of plant food allergens belongs to three groups of protein families: PR-10, LTP, and Profilins [96]. 

Among fruits who belongs to the family of *Rosaceae*, PR-10 (*Bet v* 1 family member) are the major allergens (e.g., the *Pru p* 1 in peach and *Mal d* 1 in apple) [97]. The same happens to vegetables from the *Apiaceae* family [98]: in carrots and celery, the PR-10 protein is a major allergen, especially in central Europe. These allergens are contained in both the pulp and the peel, the proteins label heat and low pH, and their synthesis is stimulated by environmental stress, as well as by the attack of pathogens [99]. These allergens usually induce only mild reactions to the oral cavity and the processing of fruits (e.g., pasteurization of juices and jams) may influence their allergenicity [96]. 

Otherwise, LTPs are small proteins with a rigid tertiary structure formed by four disulphide bridges; their function is to carry lipids through the cell walls [100]. These allergens are mainly represented in surface tissues (peel) and are in apples, peaches, apricots, cherries, plums, pears, raspberries, strawberries, blackberries, and other fruits [101]. They are stable proteins resistant to heat and acid pH; their synthesis is enhanced by the attacks of pathogens [100]. The peculiarity of these allergens may cause generalized systemic reactions in sensitized patients [102]. Recently, two types of nsLTPs have been identified from celery: a nsLTP type 1 (*Api g* 2) is expressed in the stalks and a nsLTP type 2 (*Api g* 6) is found in the tuber [103,104]. 

Populations living in the Mediterranean area are more affected by LTP sensitization compared to those living in northern Europe where sensitization to PR-10 proteins is prevalent [105,106]. The different rate of sensitization in these countries it’s related to a higher sensitization rate to *Bet v* 1 due to a relevant exposure to the pollen of Fagales tree (e.g., birch, alder, hazel) [2]. 

Profilins are small ubiquitous proteins in the plant kingdom involved in various signal transmission processes between cells and present low/intermediate stability to heat [107]. Sensitization to profilins is common, but only in few cases is it clinically relevant [108]. These allergens have been identified in apples, peaches, pears, and strawberries [109]. In vegetables, profilin is supposed to sensitize a relevant number of celery-allergic patients while in carrots it is considered a minor allergen [2]. The sensitization to profilins is equally distributed but higher in Mediterranean area [2].

Finally, Thaumatin-like proteins (TLP) have a rigid three-dimensional structure (cysteine residues forming 8 disulphide bridges) and their synthesis is stimulated by biotic and abiotic stresses [2,110]. They are allergens of apples, kiwis, peaches, and cherries [110]. They are considered minor allergens, based on data from apples, peaches, and cherries [2].

The *Bet v* 1-related food proteins, the profilins, and the nsLTPs are panallergens, with a high cross-reactivity across the plant kingdom [111]. Various clinical manifestations have been associated to these protein families, ranging from OAS to anaphylaxis. However, the prevalence of systemic reactions is higher in nsLTP-mediated fruit allergies than in the *Bet v* 1 or profilin mediated ones [2].

Based on the features of allergen families and the route of sensitization, there can be different clinical pattern of fruit allergy. Patients sensitized to trees of the family of the *Betulaceae* can develop sIgE for the *Bet v* 1-homologues of different fruits of the *Rosaceae* family [99]. Symptoms are triggered by raw food and are usually mild and localized to the oral cavity (OAS) [99].

Patients with sensitization to non-specific LTPs, mainly due to primary sensitization to peaches (*Pru p* 3 that be a sensitizer), can develop cross-sensitization to other fruits containing LTPs [101,102,112]. Clinical manifestations range from local symptoms to anaphylaxis (“LTP syndrome”) and the clinical picture can be influenced by cofactors such as alcohol, drugs, or physical exercise [113]. LTP is the major cause of food-induced anaphylaxis in Italian adults, although the proportion between the number of sensitized patients and the anaphylactic episodes patients is much lower than those observed for nuts, peanuts, or shrimp. For this reason, some authors suggest that LTP can be considered a potentially harmful yet “benign” allergen [111]. Patients sensitized to the profilins of grass pollen (*Poaceae* family) can develop a cross-sensitization to the profilin contained in the fruits of the *Rosaceae* family [114]. Despite this, sensitization to the profilin is very often clinically silent [108]. When symptomatic, the main clinical manifestation is the OAS, while the risk of systemic reaction is low [109]. 

An allergy to kiwifruit can be due to a primary sensitization process (a primary food allergy acquired through the gastrointestinal tract) or through the cross-sensitization to birch or grass pollens and latex allergens (due to cross-reactivity between *Hev b* 11, a chitinase from latex, and a homologous protein identified in kiwifruit) [2]. The allergic symptoms range from mild oropharyngeal symptoms to severe, generalized reactions. The major allergen of kiwifruit is the Actinidin (*Act d* 1), which significantly correlates with a kiwifruit’s primary sensitization [115]. Instead, sensitization to *Act d* 8 (Bet v 1- like allergen) and *Act d* 9 (profillin) is specific for patients with pollen–kiwifruit allergies [115]. The homology between kiwifruit nsLTP (*Act d* 10) and other nsLTPs is small, and therefore, there is a limited risk of cross-reactivity [116]. 

As with kiwis, other fruits (e.g., avocado, mango, chestnut, banana) show cross-reactivity with latex allergens, whose clinical manifestation is the so called “Latex-fruit syndrome” (LFS) [2]. This syndrome, firstly described in 1994, is defined as a hypersensitivity reaction to some fresh fruitsl this occurs in up to 30–50% of patients affected from a natural rubber latex (NRL) allergy and it is due to IgE antibodies that cross-react with similar epitopes on proteins phylogenetically related [117,118]. Fifteen latex allergens have been identified over the past years, named *Hev b* 1 to *Hev b* 15. Among them, four (*Hev b* 2, *Hev b* 6.02, *Hev b* 7, *Hev b* 8, and *Hev b* 11) are implicated in LFS [7,119].

## 8. Fish and Shellfish

Several allergens (stable, water soluble proteins) have been identified in seafood (fish and shellfish) and are mainly found in the edible meat [120].

### 8.1. Fish

Fish belongs to the Phylum of Chordata [121]. Fish allergens have been identified in many parts of the fish such as muscle, skin, bones, roe, milt, and blood. Parvalbumins are the major allergens of fish and are resistant to heat and to enzymatic digestion (Table 7). Parvalbumin is a small protein contained in the muscle of several fishes including cod (*Gad c* 1), salmon (*Sal s* 1), carp (*Cyp c* 1), tuna (*Thu a* 1), swordfish (*Xip g* 1), and pilchard (*Sar s* 1). Further, it is involved in up to 70–100% of fish-induced allergic reactions. Moreover, parvalbumins show a high degree of identity and patients sensitized to the parvalbumin of one fish may present allergic reaction to parvalbumines contained in other fishes [5]. Minor fish allergens include aldolase Alfa and Beta-enolase (which are expressed in fish muscle from cod (*Gad m* 2, *Gad m* 3), salmon (*Sal s* 2, *Sal s* 3), and tuna (*Thu a* 2, *Thu a* 3)), fish gelatin (collagen), and vitellogenins [2].

### 8.2. Shellfish (Crustaceans and Molluscs)

Crustaceans (i.e., crabs, lobsters, crayfish, and shrimp) belong to the phylum of *Arthropoda* [121]. Above them, shrimps are widely consumed: they belong to the family of the *Penaeidae,* which includes the giant freshwater shrimp (*Macrobrachium rosenbergii*), the royal shrimp (*Melicertus latisulcatus*), the Indian shrimp (*Penaeus indicus*), the gulf brown shrimp (*Penaeus aztecus*), the northern prawn (*Pandalus borealis*), and the giant shrimp (*Penaeus monodon*) [121,122]. 

The allergenic components are mainly localized in the cephalothorax, muscle tissue, and eggs. Their function is essential for movement and energetic metabolism [122]. The main allergenic proteins contained in different shrimp species include tropomyosin (e.g., *Pen a* 1, *Pen m* 1, *Pen i* 1, *Mac r* 1, *Mel l* 1), arginine kinase (ex. *Pen m* 2), troponin C (e.g., *Pen m* 6), the light chain 2 of the myosin (e.g., *Pen m* 3), and calcium-binding proteins (e.g., *Pen m* 4) (Table 7) [2,122]. 

Among these, the most studied is tropomyosin as it represents the panallergen of crustaceans. This allergen belongs to a family of highly conserved structural proteins, stable to heat, and involved in muscular contraction. These have a high degree of amino acid sequence identities not only among the different species of crustaceans but also among crustaceans and molluscs, mites, and other invertebrates [123]. Tropomyosin is also considered to be a major allergen of shrimps and crustaceans and represents a marker of food allergy: 72–98% of the subjects allergic to shrimps has IgE specific for tropomyosin. Sensitization towards tropomyosin increases the risk of reaction to OFC in subjects with suspected shellfish allergy [124]. This allergen is also implicated in the mechanisms of cross-reactivity between dust mites and shellfish. Up to 90% of shrimp allergic subjects also have sIgE for the mites, as the presence of cross reactivity to the dust tropomyosin *Der p* 10 is a minor allergen of *D. Pteronyssinus* [123]. 

The allergy work-up in patients with a suspected seafood allergy always includes reviewing an accurate history, executing tests in vivo diagnostics, investigating the tolerance to crustaceans/molluscs and fish and the presence of respiratory allergies in particular asthma, and detecting sIgE for implicated and cross-reactive allergens and for the available molecular components [2]. 

## 9. Mammalian Meat

Allergies to mammals-derived meat is infrequently considered, since it is primarily a disease of young atopic children with allergic reactions that occurred rapidly after exposure [125,126]. In the last few years, new meat allergy entities have been recognized (Table 8), which interested predominantly adults and can present delayed-onset reactions [125,127]. 

Cows are the only species with a significant number of recognized food allergens: up to now nine allergenic proteins have been identified as food allergens [125]. However, most of these allergens have been initially identified as allergens in CM [125], since the majority of the reported reactions to beef occurred in CM-allergic children [128]. Data from literature shows that approximately 10% of CM-allergic children have a clinical reaction after eating beef [129]. Although the major allergens of beef are both BSA (*Bos d* 6) and immunoglobulin IgG (*Bos d* 7), the first seems to be the most relevant allergen in these reactions [125,130]. Thus, in the diagnostic work-up for CM-allergic children, the study of sIgE to *Bos d* 6 could be relevant to identify patients at risk of beef-induced reactions.

Another particular form of meat allergy is represented by the so-called “cat-pork syndrome” [131], where sensitization to domestic furry mammals (usually cats) can induce IgE-mediated hypersensitivity reactions after eating pork meat [125]. This reaction can be based on cross-reactive serum albumins (66–69 kDa) from mammals as *Fel d* 2 in acts and the pork meat allergen *Sus s* 6 [132]. *Fel d* 2 is a 67-kDa serum albumin and is a minor cat allergen, against whom only about 15–35% of cat allergic subjects are sensitized [132]. Considering that about 30% of patients sensitized to *Sus s* 6 show allergic reactions after pork meat ingestion, only 1–3% of cat-allergic patients seem to be at risk for an allergy to pork meat [132]. 

Recently, a delayed allergic reaction after eating mammalian meat has been described. This particular type of IgE mediated allergy is attributable to a new relevant carbohydrate allergen galactose-alpha-1,3-galactose (*Alpha-gal*), whose sensitization is triggered by tick bites [129]. 

The identification of *Alpha-gal* was based on the observation that patients suffered from severe anaphylaxis upon first exposure to the monoclonal antibody cetuximab [133]. The analysis of IgE antibodies to cetuximab showed that these antibodies were specific for oligosaccharide residues on the heavy chain and *Alpha-gal* was identified as the relevant epitope [134]. *Alpha-gal* is a glycan of non-primate mammals that is homologous to the B-group blood antigen and is present on all forms of tissue and products derived from mammals including red meat, kidney, gelatin, milk, cheese, and gelatin-containing vaccines [123,124]. The presence of sIgE in *Alpha-gal* was associated with episodes of delayed angioedema urticaria and anaphylaxis after the ingestion of red meat [135,136]. Sensitized subjects can react to all the products containing *Alpha-gal*. Recently, this was described in an episode of anaphylaxis after vaccination containing gelatin was given to a sensitized pediatric patient [137]. 

The diagnosis of this allergy can be difficult because there is often a delay a 3–6 h window between eating mammalian meat and the appearance of symptoms. Most patients develop this allergy after many years of safely eating beef or pork meat [126,136]. Recent data suggests that subjects with B-group blood antigens are protected from developing *Alpha-gal* sensitization [138].

The immunologic mechanism that contributes to sensitization and to delayed symptoms is still not clear. To date, the only known route for sensitization is by tick bites [125]. Three different tick species are implicated: *Amblyomma Americanum* (USA), *Ixodes Holocyclus* (Australia), and *Ixodes Ricinus* (Europe) [125]. The evidence supporting the role of tick bites in the sensitization process to *Alpha-gal* is various: four cases were described with an epidemiologic evidence that sIgE to *Alpha-gal* increased following documented tick bites. Moreover, sIgE for *Alpha-gal* were found in areas where tick bites are common and the global distribution of delayed anaphylactic reaction to red meat is similar to the distribution of the various tick species [139]. Indeed, Hamsten et al. showed that *Alpha-gal* is present in the gastrointestinal tract of *Ixodes Ricinusn;* this can cause host exposure to *Alph-gal* during a tick bite [140]. 

In summary, the development of sIgE to *Alpha-gal* is an emerging cause of food allergy and anaphylaxis after ingestion of meat that commonly emerges during adulthood but can also be present in children. Moreover, it is characterized by a delayed onset of symptoms, a red meat free diet, and is related to a preceding tick bite [126,136]. 

We suggest future studies consider the possible presence of IgE-mediated allergy against the *Alpha-gal* in case of urticaria, angioedema, or anaphylaxis, which arise at 3–6 h from the intake of red meat.

## 10. Conclusions

The advent of CRD represents a milestone in the field of food allergy diagnosis, allowing for a better identification and characterization of the specific molecules that trigger allergic reactions. In light of this, CRD has become an important tool in the diagnostic work-up of food allergies, given the identification of sIgE against the major allergens allows for discriminating against primary food allergies versus secondary sensitization. Moreover, CRD helps predict the evolution of the allergic process and the clinical risk of each patients and in stratifying the outcome of the OFC. 

Despite this, up until now there are still many gaps both in the research area and at the clinical level. First, only some of the most relevant allergens are available for commercial diagnostic assays. Second, CRD is a relatively expensive assay when compared with the first and second level diagnostic tests (SPT and allergen extract-based sIgE). Third, CRD has not shown a level of specificity and sensitivity as optimal as to become the gold standard in the diagnosis of food allergy for the identified allergens; this still remains the OFC. Further research and future efforts should be addressed to fill these gaps.

## Figures and Tables

**Table 1:** Molecular allergens identified in cow’s milk. Allergens available for diagnostics are marked in bold.

Cow’s Milk Protein	Allergen	Allergenicity	Features
Casein Family (coagulum: has approximately 80% of the CM proteins)			
Casein	**Bos d 8**	Major	Resistant to high temperatures High sequence homology (>85%) with proteins from goat and sheep Very low cross- reactivity (<5%) with milks from donkey, mare, buffalo, or camel
Alpha s1-casein	*Bos d 9*	Major	
Alpha s2-casein	*Bos d 10*	Major	
Beta-casein	*Bos d 11*	Major	
Kappa-casein	*Bos d12*	Major	
Whey (sensible to heating, lose IgE binding after 15–20 min of boiling at >90 °C) [16]			
Alpha-lactalbumin	**Bos d 4**	Major	~65% of whey, present in the milk of almost all mammals
Beta-lactoglobulin	**Bos d 5**	Major	~25% of whey, not present in the human breast milk
Bovine serum albumin	**Bos d 6**	Minor	~8% of whey, is one of the major beef allergens, responsible for cross reactivity between CM and raw beef
Immunoglobulins	*Bos d 7*	Minor	Especially G class, may play a role in cross-reactivity with beef [8]
Lactoferrin	**Bos d lactoferrin** *	Minor	Is a multifunctional protein of the transferrin family [8]

* Available only for semiquantitative methods.

**Table 2:** The molecular allergens available for component resolved diagnosis for hen eggs.

Hen Eggs Allergen Name	Features
*nGal d 1* (Ovomucoid)	White-serine protease inhibition activity with high resistance to heating and chemical denaturation Highly allergenic, correlated to high risk for reaction to all forms of egg
*nGal d 2* (Ovalbumin)	Serine protease inhibitor, heat-labile it is the most abundant egg white protein It is correlated with risk for clinical reaction to raw or slightly heated egg and certain vaccines
*nGal d 3* (Conalbumin)	low resistance to heating and chemical denaturation
*nGal d 5* *	Hen yolk/chicken meat

* Available only for semiquantitative methods.

**Table 3:** Soybean molecular allergens available for component resolved diagnosis.

Soybean Allergen Name	Biochemical Name and Features	
*rGly m 4*	PR-10	Cross-reactive allergen Reactions in Birch allergic patients
*nGly m 5* (Beta conglycinin)	7S Globuline	Major allegens Implicated in primary sensitization Severe reactions
*nGly m 6* (Glycinin)	11S Globuline	

**Table 4:** The molecular allergens available for component resolved diagnosis for peanuts and tree nuts.

Allergen Source	Biochemical Name		
	Stable Proteins		Labile Proteins
	SSP	LTP	PR-10
Peanut *Arachis hypogaea*	r*Ara h* 1 r*Ara h* 2 r*Ara h* 3 r*Ara h* 6	r*Ara h* 9	r*Ara h* 8
Hazelnut *Corylus avellana*	r*Cor a* 9 r*Cor a* 14	r*Cor a* 8	r*Cor a* 1
Cashew nut *Anacardium occidentale*	r*Ana o* 3 r*Ana o* 2 *		
Walnut *Juglans regia*	r*Jug r* 1 n*Jug r* 2 *	r*Jug r* 3	
Brazil nut *Bertholletia axcelsa*	r*Ber e* 1		

* Available only for semiquantitative methods.

**Table 5:** Wheat molecular allergens available for component resolved diagnosis.

Allergen Name	Biochemical Name	Molecular Weight (kDa)	Clinical Relevance
*Tri a* 14	Non-specific LTP 1	9	Food allergen in Italian patients Baker’s asthma
*Tri a* 19	ω-5 gliadin	65	Food allergy in children WDEIA
n*Tri aA*_TI *	Alpha-amylase inhibitors	13	Food allergy

* Available only for semiquantitative methods.

**Table 6:** Plant foods molecular allergens available for component resolved diagnosis.

Fruit/Vegetable Source	Biochemical Name					
	Actinidin	LTP	Kiwellin	TLP	PR-10	Profilin
Apple *Malus domestica*		r*Mal d* 3			r*Mal d* 1	
Kiwi *Actinidia deliciosa*	n*Act d* 1 *		n*Act d* 5 *	n*Act d* 2 *	r*Act d* 8	
Peach *Prunus persica*		r*Pru p* 3			r*Pru p* 1	r*Pru p* 4
Celery *Apium graveolens*					r*Api g* 1.01	

* Available only for semiquantitative methods.

**Table 7:** Seafood molecular allergens available for component resolved diagnosis.

Seafood Source	Allergen Name	Biochemical Name	Features
Carp	r*Cyp c* 1	Parvalbumin	Major allergen sIgE (sIgE) are suggestive of true fish allergy
Cod	r*Gad c* 1	Parvalbumin	Major allergen sIgE are suggestive of true fish allergy
Shrimp	r*Pen a* 1	Tropomyosin	Major allergen sIgE are suggestive of true crustaceans allergy cross-reacts with tropomyosin of mites
	n*Pen m* 2 *	Arginine kinase	Minor allergen
	n*Pen m* 4 *	Calcium binding protein	Minor allergen

* Available only for semiquantitative methods.

**Table 8:** Diagnosis of meat allergic reactions. Modified from [111].

Type of Meat Allergy	History	IgE	Major Allergen
Primary meat sensitivity in childhood	Immediate reactions to meat Often with pre-existing sensitivity to cow’s milk	Milk Relevant meat	*Bos d* 6
Pork–Cat Syndrome	Reactions to pork within 1 h. In some cases with additional reactions to beef In most cases pre-existing sensitization to cats	Pork Cat Beef Porcine	*Fel d* 7 Sus s 6
Delayed Anaphylaxis to Red Meat or the Alpha-Gal syndrome	Urticaria and/or anaphylaxis occurring 3–6 h after eating beef	Beef Lamb Pork	*Alpha-gal*
